# Selective uptake of boronated low-density lipoprotein in melanoma xenografts achieved by diet supplementation.

**DOI:** 10.1038/bjc.1996.618

**Published:** 1996-12

**Authors:** Y. Setiawan, D. E. Moore, B. J. Allen

**Affiliations:** Department of Pharmacy, University of Sydney, NSW, Australia.

## Abstract

The lipid core of human plasma low-density lipoprotein (LDL) was extracted using hexane and the LDL reconstituted with the addition of n-octyl-carborane. Biodistribution studies of the boronated LDL were performed in BALB/c mice bearing subcutaneous Harding-Passey melanoma xenografts. When diet supplementation with coconut oil and cholesterol for 21 days and regular dosing with hydrocortisone for 7 days before the studies was used to down-regulate the liver LDL receptors and the adrenal receptors, respectively, the tumour-blood boron concentration ratio of 5:1 was achieved.


British Journal of Cancer (1996) 74, 1705-1708
© 1996 Stockton Press All rights reserved 0007-0920/96 $12.00

SHORT COMMUNICATION

### Selective uptake of boronated low-density lipoprotein in melanoma xenografts achieved by diet supplementation

Y Setiawan[1], DE Moore[1] and BJ Allen[1,2]

[1]Department of Pharmacy, The University of Sydney, Sydney, NSW 2006; [2]St George Cancer Care Centre, Sydney, NSW 2217, Australia.

**Summary** The lipid core of human plasma low-density lipoprotein (LDL) was extracted using hexane and the LDL reconstituted with the addition of *n*-octyl-carborane. Biodistribution studies of the boronated LDL were performed in BALB/c mice bearing subcutaneous Harding–Passey melanoma xenografts. When diet supplementation with coconut oil and cholesterol for 21 days and regular dosing with hydrocortisone for 7 days before the studies was used to down-regulate the liver LDL receptors and the adrenal receptors, respectively, the tumour–blood boron concentration ratio of 5:1 was achieved.

**Keywords:** low-density lipoprotein; boron neutron capture therapy; melanoma xenograft; boron compound pharmacokinetics; reconstituted low-density lipoprotein; boronated low-density lipoprotein

The concept of using plasma low-density lipoprotein (LDL) as a cancer-targeted drug carrier system has been stimulated by the discovery that leukaemic cells from the peripheral blood of patients with acute myelogenous leukaemia had 3–100 times higher LDL receptor activity than normal white blood cells (Ho *et al.*, 1978). Since then, a number of studies have demonstrated that a variety of human cancers, including cervical, lung and brain tumours, take up LDL at a rate greater than the corresponding normal tissue (for a review see Firestone, 1994).

The adaptation of LDL as a boron carrier for boron neutron capture therapy (BNCT) of cancer has been pioneered by Kahl and co-workers, who described the extraction of the cholesterol ester core and reconstitution with various carborane compounds (Kahl and Callaway, 1989). The boronated LDL retained its ability to be taken up strongly into hamster V79 and CHO cells in culture, achieving boron concentrations consistent with a receptor-mediated binding mechanism (Laster *et al.*, 1991). Subsequent *in vitro* neutron irradiation confirmed the efficacy of the boron neutron capture reaction in killing the cells. However, *in vivo* studies of boronated LDL with hamster V79 and CHO cell xenografts in mice did not show selective localisation of boron in the cells (Kahl *et al.*, 1993).

In this paper, it is shown for the first time that boronated LDL can act as a novel boron delivery system in BNCT, following suppression of the non-autonomous LDL receptors.

### Materials and methods

### *Preparation of boronated LDL*

Human LDL (density 1.019–1.063 g $ml^{-1}$) was isolated by two-step density ultracentrifugation from fresh human plasma using standard procedures (Havel *et al.*, 1955). The boronated LDL was prepared by extracting the lipid core of LDL with heptane, then reconstituting according to the method of Masquelier *et al.* (1986) with *n*-octyl [$^{10}$B]-carborane, which was synthesised in our laboratory (Smith *et al.*, 1996). Briefly, the procedure was as follows. LDL (300 $\mu$l containing 2 mg of LDL protein) was transferred into a silanised glass tube (13 x 100 mm). The LDL was lyophilised in the presence of sucrose (25%, w/v) as cryoprotectant for 6 h, then extracted three times with 5 ml heptane at 4°C with repeated agitation every 10 min for 1 h. *n*-Octyl carborane (20 mg in 1 ml heptane) was combined with the dried heptane extract and mixed with the extracted LDL by gentle agitation at 4°C. After 1 h, the heptane was evaporated under nitrogen and the drug–LDL complex was solubilised by the addition of 1 ml of 10 mM tricine buffer pH 8.4, and left overnight at 4°C. Insoluble non-incorporated drug was separated by centrifuging at 4°C for 5 min in an Eppendorf centrifuge and, finally, the boronated LDL preparation was filtered through a 0.45 $\mu$m membrane filter.

### *Pharmacokinetics of the boronated LDL drug carrier system in melanoma-bearing mice*

Adult male BALB/c mice at 6–8 weeks of age weighing 25–30 g were housed under a normal light–dark cycle, and provided with diet and water *ad libitum*. One group of mice was fed Gordon's Pelletised Animal Feed (Brisbane, Australia), while the remainder were fed for 3 weeks before the pharmacokinetic study with the same feed supplemented with 2% (w/w) cholesterol and 10% (w/w) coconut oil (cholesterol was dissolved in melted coconut oil at 100°C and mechanically mixed into the pelletised animal feed). This diet was employed to down-regulate the non-autonomous liver LDL receptors. In addition, to down-regulate the adrenal LDL receptors, a daily intraperitoneal (i.p.) injection of hydrocortisone sodium succinate (5 mg per mouse) was given for 7 days before each study (Ponty *et al.*, 1993). The oil-enriched feed was continued throughout the studies. In 3 weeks the mice (initial weight $17.67 \pm 0.53$ g, $n=20$) had gained an average of $3.3 \pm 1.2$ g ($n=10$) in weight when 'diet-supplemented', compared with a weight gain of $3.0 \pm 1.3$ g ($n=10$) for the mice fed the normal diet for the same period, a difference which is not significant ($P=0.19$).

Tumour induction in the BALB/c mice was achieved by inoculating 0.1 ml of medium containing $10^6$ Harding–Passey melanoma cells subcutaneously in the right thigh. After a period of 7 days the tumours had grown to an approximate diameter of 2 mm (20–70 mg) and the mice were then injected i.p. with 1 ml of boronated LDL, equivalent to 4 mg of *n*-octyl carborane. At different times the mice were sacrificed and tissue samples of 20–100 mg (blood, liver, kidney, spleen, brain, tumour and skin and muscle adjacent to the tumour) were collected for each animal. The samples were weighed directly into polythene vials and digested in a water bath for 1 h at 70°C with 0.5 ml of perchloric acid (70%) and 1 ml of hydrogen peroxide (28%) (Tamat *et al.*, 1987). An internal standard solution of

Correspondence: DE Moore
Received 3 November 1995; revised 11 January 1996; accepted 12 June 1996

beryllium-9 (Spectrosol, beryllium sulphate from BDH) was added to each sample to give a final $^9$Be concentration of 50 p.p.b. After the vial contents became colourless, the sample was cooled, and 8.3 ml deionised water was added. The sample was filtered through a 0.45 $\mu$m membrane filter before being analysed for boron content by inductively coupled plasma mass spectrometry (Perkin-Elmer FIAS 440).

### Results

### *Physical characterisation of boronated LDL*

The LDLs from which the cholesterol ester core had been extracted, and then reconstituted with *n*-octyl-carborane, were identical in physical characteristics to the native LDLs. Capillary electrophoresis showed that both boronated and native LDLs have similar electrophoretic mobility, suggesting that the incorporation of the carborane compound into the LDLs did not alter the characteristics of the apoprotein-B100. Additionally, negatively stained electron micrographs showed that boronated and native LDLs have identical morphology and a uniform size of around 20 nm in diameter.

### *Quantification of boron incorporation into LDL*

The total protein concentration of the LDL as measured by the Coomassie blue dye-binding assay (Bradford, 1976), varied from batch to batch in the range 15–25 mg ml$^{-1}$. The boron content was measured using infrared spectrometry after extraction in carbon tetrachloride solution (Setiawan *et al.*, 1994). As administered in the pharmacokinetic experiments, 1 ml of the LDL preparation contained 4 mg of *n*-octyl-carborane (corresponding to 1.7 mg boron) in 20.2 mg apo-B100, i.e. 19.7% incorporation in respect to the apo-B100.

### *Pharmacokinetics of the boronated LDL drug carrier system*

Two biodistribution studies were performed. In study A the pharmacokinetic parameters of boronated LDL in tumour-bearing mice were determined using four mice per time point (two fed the regular diet and two diet-supplemented mice). In study B the biodistribution, in diet-supplemented mice, of boronated-LDL was compared with that for the same boron compound dissolved in arachis oil, in order to ascertain the role of the LDL as a tumour delivery vehicle. Pure *n*-octyl-carborane was dissolved in 200 $\mu$l of arachis oil. The same boron dose (4 mg *n*-octyl-carborane, equivalent to 1.7 mg boron) was given i.p. in all cases.

*Study A* While diet supplementation had no significant effect on the weight of the mice, it was responsible for a marked effect on the uptake of boronated LDL by the liver and the tumour, as shown in Figure 1a and b. Boron concentration in the liver was reduced by about 30% from 2 to 4 h after administration, but was of similar magnitude at 7 h. On the other hand, the boron concentration in the tumour increased markedly to about 5 p.p.m. at 12 h, compared with 1 p.p.m. at the same time in the control mice ($P = 0.0043$). There was no observable difference in the boron distribution in the skin and muscle adjacent to the tumour site. The boron concentration was also determined in the kidney, spleen and brain, but, for clarity, these are not shown in Figure 1. The kidney and spleen values followed the same trend as the liver, but were about half the magnitude, while brain uptake of boron was less than in the skin/muscle samples.

The pharmacokinetic data shown in Table I illustrate the profound changes arising from the diet supplementation. The elimination constant ($k_{el}$) and half-life ($t_{1/2}$) of the boron concentration in the blood following administration of B-LDL to normal (group ND) and diet-supplemented (group DS) mice were not significantly different. The volume of distribution ($V_{D(ss)}$) and plasma clearance ($Cl_p$) of boronated LDL in group ND was higher than group DS, but the area under the plasma boron concentration curve (AUC) for group ND was lower than for group DS. These results suggest that diet supplementation with coconut oil and cholesterol does not change the metabolism of the boronated LDL, so that the $k_{el}$ and $t_{1/2}$ do not change. However, the diet supplementation does down-regulate the liver and adrenal gland uptake of boronated LDL, so that the volume of distribution and the plasma clearance decreased for group DS, and consequently the area under the curve increased.

**Figure 1** Study A. Boron concentration in parts per million measured in various tissues of mice bearing Harding–Passey melanoma xenografts, at varying times after administration of reconstituted LDL (1 ml) containing 4 mg of *n*-octyl carborane and 20.2 mg apo-B protein (two mice at each time point). (**a**) Mice were fed a normal diet (group ND). (**b**) The mice were pretreated with a cholesterol-rich diet for 3 weeks and hydrocortisone injections for 1 week before tumour cell inoculation (group DS).

**Table I** Pharmacokinetic parameters for the biodistribution of boronated low-density lipoprotein in tumour-bearing mice fed normal and oil-supplemented diets

| *Pharmacokinetic parameter* | *Group ND (normal diet)* | *Group DS (diet supplemented)* | *Units* |
|---|---|---|---|
| AUC | 22.45±0.52 | 45.08±1.7 | $\mu$g ml$^{-1}$ h |
| $Cl_p$ | 178.2±4.1 | 88.7±3.4 | ml h$^{-1}$ |
| $k_{el}$ | 0.12±0.01 | 0.13±0.01 | h$^{-1}$ |
| $t_{1/2}$ | 5.54±1.13 | 5.44±0.21 | h |
| $V_{d(ss)}$ | 1600±37 | 861±33 | ml |

**Figure 2** Study B. Boron concentration in parts per million measured in various tissues of mice bearing Harding–Passey melanoma xenografts, at varying times after administration of (**a**) reconstituted LDL (1 ml) containing 4 mg of *n*-octyl-carborane and 20.2 mg apo-B-protein (three mice at each time point); or (**b**) arachis oil (0.2 ml) containing 4 mg of *n*-octyl-carborane (two mice at each time point). All mice were pretreated as described for Figure 1b. ●, Blood; □, liver; ▽, skin/muscle; ▼, tumour.

*Study B* The pharmacokinetics of boron uptake for boronated-LDL and the carborane in arachis oil for diet-supplemented mice are quite different, as shown in Figure 2a and b. As in study A, only the data for blood, liver, tumour and skin/muscle are shown. The boronated LDL was distributed as in study A, with a broad maximum boron concentration in the tumour of $3.5 \pm 1.2$ p.p.m. at 18 h, whereas there is no significant time dependence when the carborane was administered in oil. The 18 h maximum uptake in the tumour using boronated-LDL is similar to that obtained by Ponty *et al.* (1993) after administration of $^{99m}$Tc-labelled LDL to B16 melanoma-bearing CD57BL/6J mice. The tumour–blood ratio is about 5 for boronated-LDL, whereas it is about 2 for the carborane in oil. Further, the liver boron uptake is much higher for the boronated LDL.

### Discussion

The most significant finding from the biodistribution studies of boronated LDL in mice is that down-regulation of both liver LDL receptors, by a diet supplemented with coconut oil and cholesterol, and adrenal receptors, by regular dosing with hydrocortisone, increased the selective uptake of boronated LDL by subcutaneous Harding–Passey melanoma xenografts in BALB/c mice, achieving a tumour–blood boron concentration ratio of 5:1. The selectivity of the boronated LDL to the tumour cannot be ascribed to the boron compound, but to the LDL itself. This result clearly shows for the first time that the LDL apoprotein retains its *in vivo* receptor recognition character through the process of boron compound incorporation.

In principle, the use of boronated LDL can be optimised further by i.v. administration of a more concentrated preparation and further down-regulation of the LDL receptors, so that the potential to achieve therapeutic concentrations of boron in the tumour (>20 p.p.m. boron in the tumour) leading to successful control with neutron irradiation may be realised.

In current trials of neutron capture therapy, brain tumours are the clinical target, so the question arises as to the potential of boronated LDL to deliver boron to the brain. Normal brain is not expected to allow the passage of these large entities, and our results showed clearly that brain boron concentrations were less than that in blood. However, other studies have shown that several different types of brain tumour bind two to three times more LDL than normal brain, indicating that the blood–brain barrier may be significantly more permeable around the tumour (Rudling *et al.*, 1990). On this basis, the further investigation of LDL for boron delivery in BNCT is warranted.

**Acknowledgements**
The assistance of Michael Smith and Julia Mallesch is gratefully acknowledged. This work was supported in part by the Australian Institute of Nuclear Science and Engineering and the Australian Research Council.

### References

BRADFORD MM. (1976). A rapid and sensitive method for the quantitation of microgram quantities of protein utilising the principle of protein-dye binding. *Anal. Biochem.*, **72**, 248–254.

FIRESTONE RA. (1994). Low density lipoprotein as a vehicle for targetting antitumour compounds to cancer cells. *Bioconjug. Chem.*, **5**, 105–113.

HAVEL RJ, EDER HA AND BRAGDON JH. (1955). The distribution and chemical composition of ultracentrifugally separated lipoproteins in human serum. *J. Clin. Invest.*. **34**, 1345–1353.

HO YK, SMITH RG, BROWN MS AND GOLDSTEIN JL. (1978). Low density lipoprotein (LDL) receptor activity in human acute myelogenous leukemia cells. *Blood*, **52**, 1099–1104.

KAHL SB AND CALLAWAY JL. (1989). New tumour localizer: advances in the use of low density lipoproteins (LDL). *Strahlenther. Onkol.*, **165**, 137–140.

KAHL SB, WAINSCHEL LA, PATE DW, CALLAWAY JC AND HILTUNEN J. (1993). *In vitro* and *in vivo* studies with boronated low density lipoproteins for neutron capture. In *Proc. Clinical BNCT Workshop Helsinki.* Autrinen L and Kallio M. (eds) pp. 105–109.

LASTER BH, KAHL SB, POPENOE EA, PATE DW AND FAIRCHILD RG. (1991). Biological efficacy of boronated low density lipoprotein for boron neutron capture therapy as measured in cell culture. *Cancer Res.*, **51**, 4588–4593.

MASQUELIER M, VITOLS S AND PETERSON C. (1986). Low-density lipoprotein as a carrier of antitumoral drugs: *in vivo* fate of drug–human low-density lipoprotein complexes in mice. *Cancer Res.*, **46**, 3842–3847.

PONTY E, FAVRE G, BENANIBA R, BONEU A, LUCOT H, CARTON M AND SOULA G. (1993). Biodistribution study of $^{99m}$Tc-labelled LDL in B16 melanoma bearing mice. Visualization of a preferential uptake by the tumor. *Int. J. Cancer*, **54,** 411–417.

RUDLING MJ, ANGELIN B, PETERSON CO AND COLLINS VP. (1990). Low density lipoprotein receptor activity in human intracranial tumours and its relation to cholesterol requirement. *Cancer Res.*, **50,** 483–487.

SETIAWAN Y, RISE T AND MOORE DE. (1994). Fourier transform infrared (FTIR) spectrometry for the assay of polyhedral boron compounds in plasma and pharmaceutical formulations. *Pharmaceut. Res.*, **11,** 723–727.

SMITH MD, SETIAWAN Y AND MOORE DE. (1996). Optimisation of drug anchor for *ortho*-carborane in reconstitution of low density lipoprotein. In *Cancer Neutron Capture Therapy*, Mishima Y (ed.) pp.143–149. Plenum Press: New York.

TAMAT SR, MOORE DE AND ALLEN BJ. (1987). Assay of boron in biological tissues by inductively coupled plasma atomic emission spectrometry. *Anal. Chem.*, **59,** 2161–2164.


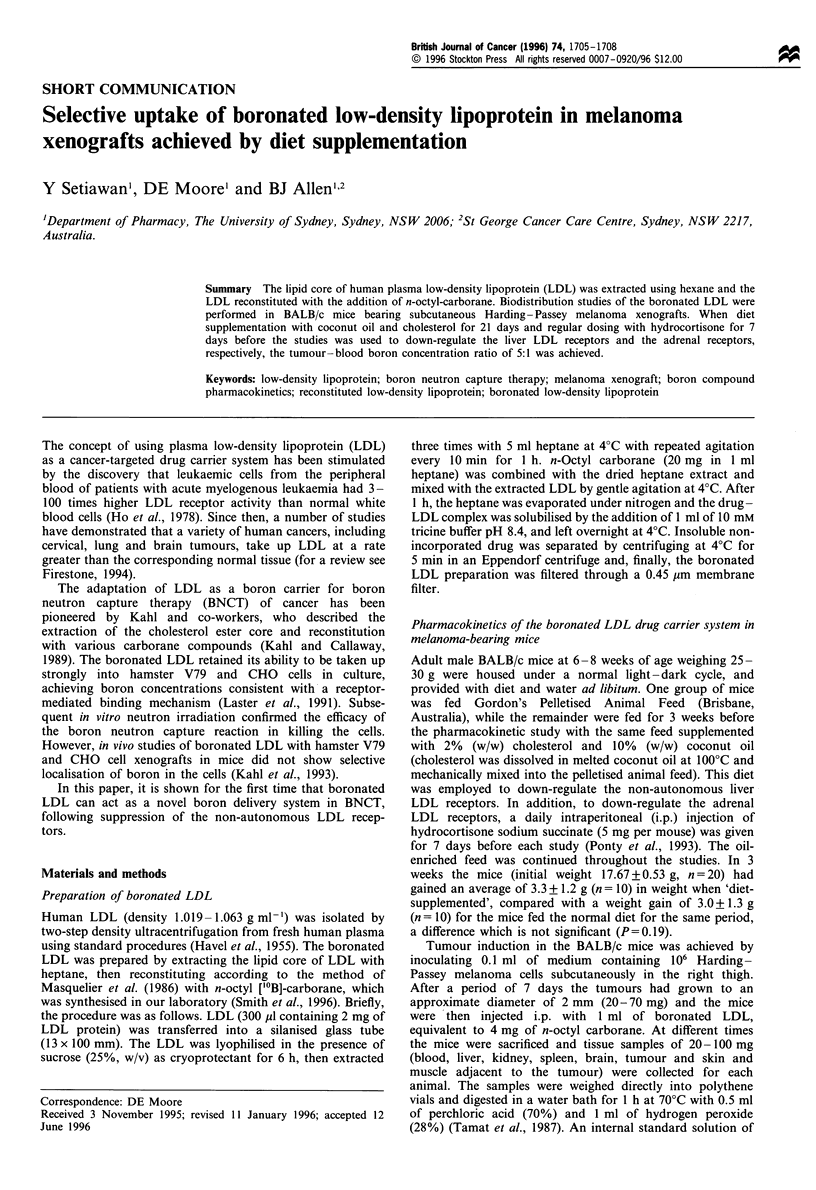

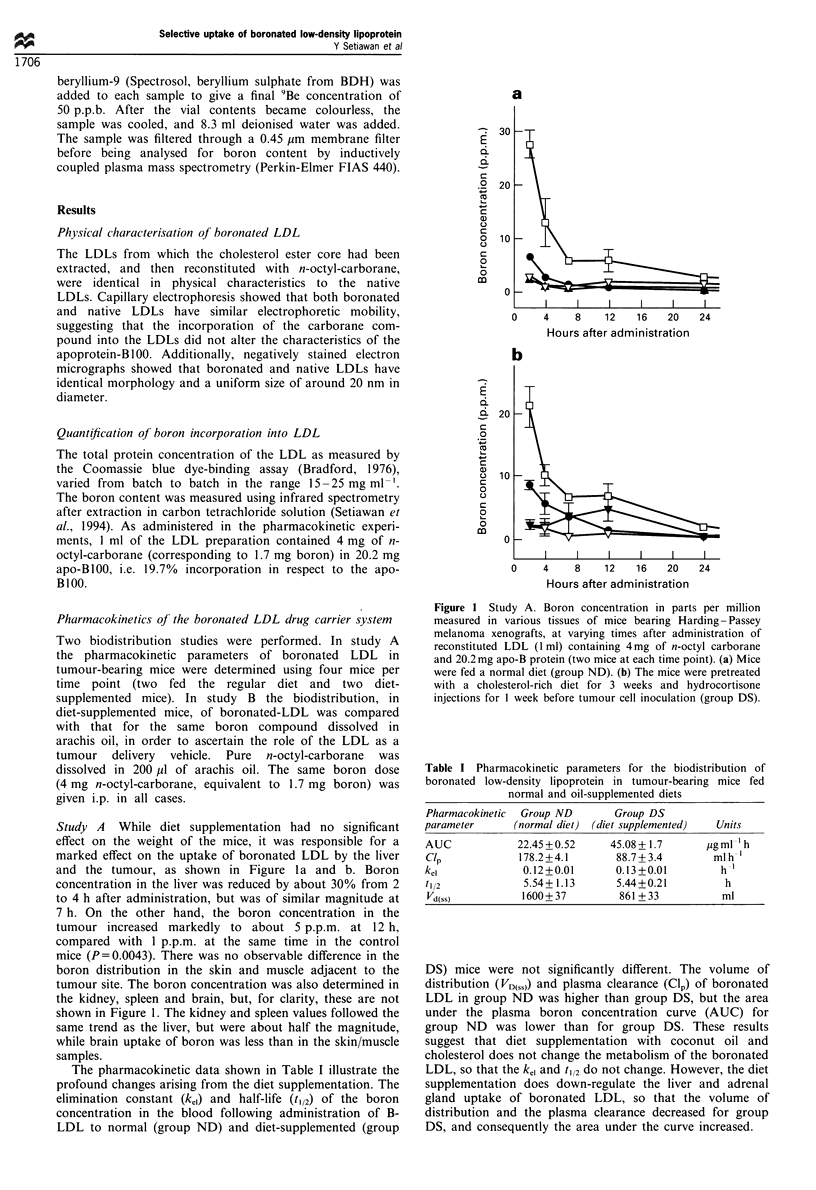

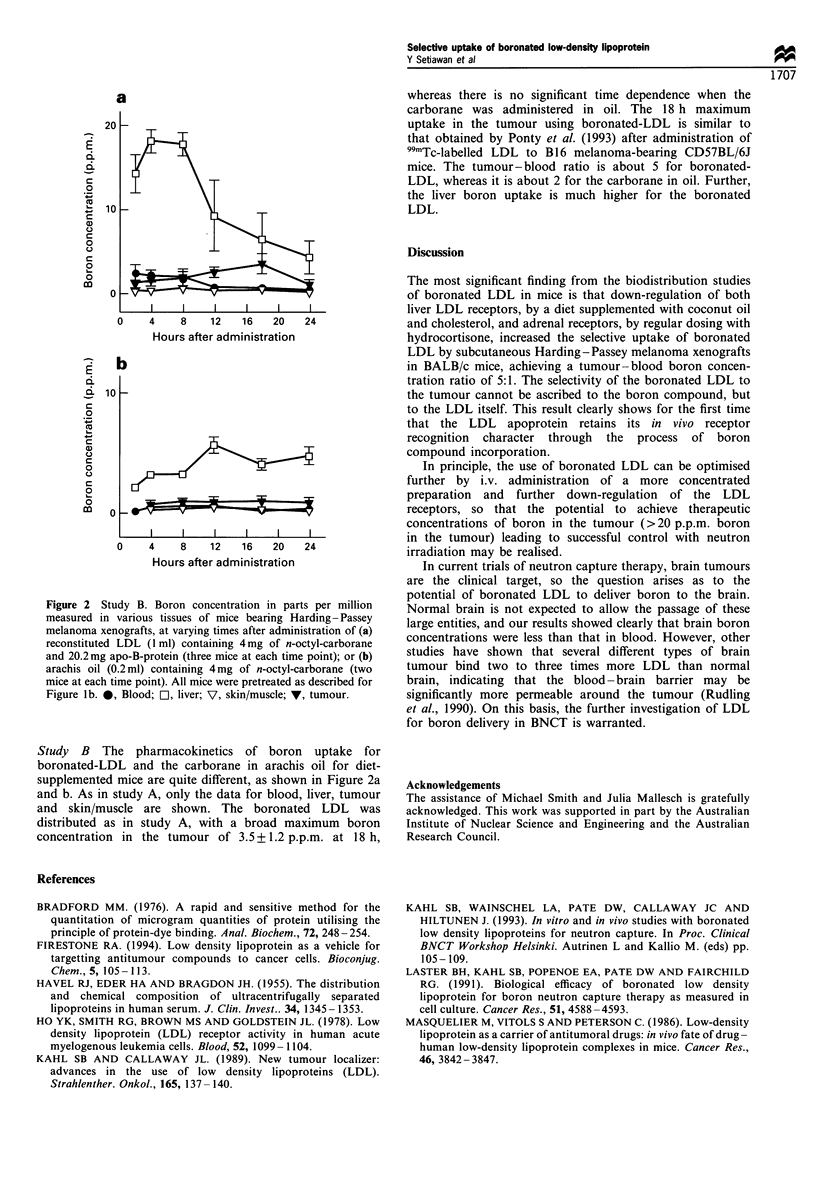

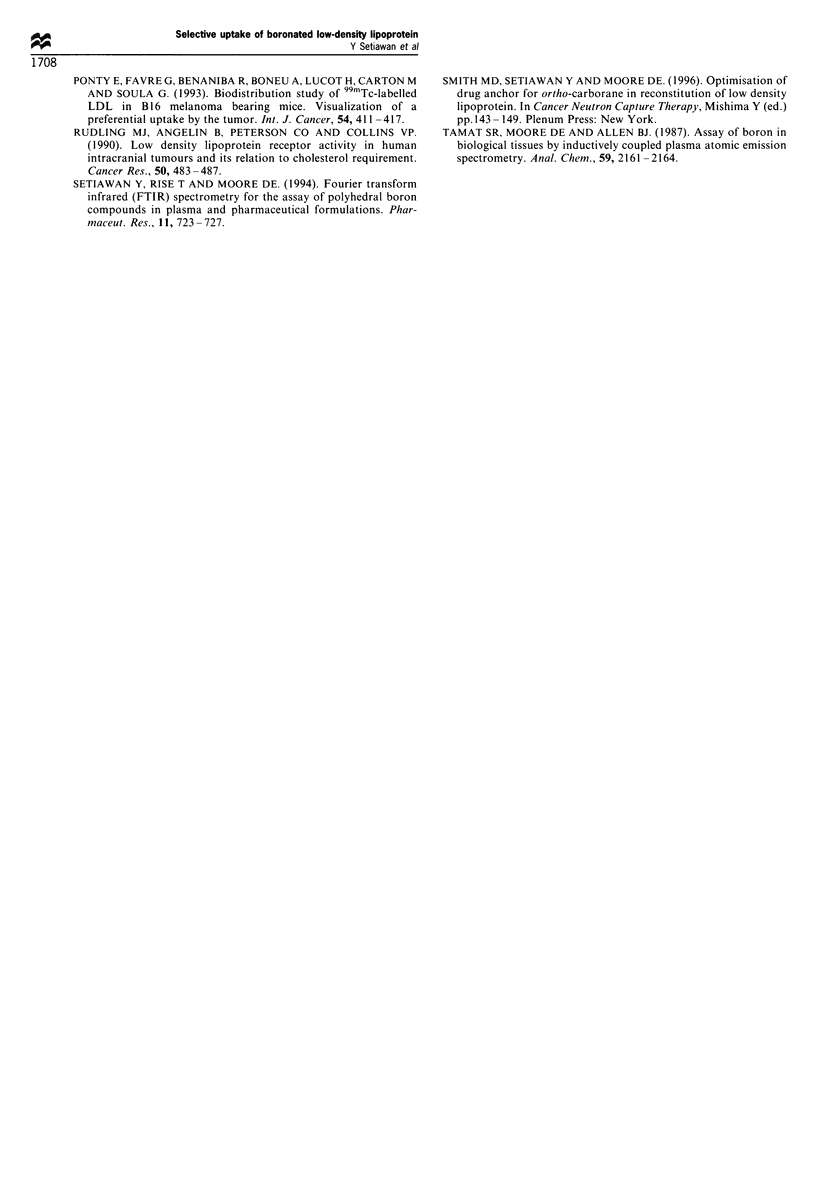


## References

[OCR_00442] Bradford M. M. (1976). A rapid and sensitive method for the quantitation of microgram quantities of protein utilizing the principle of protein-dye binding.. Anal Biochem.

[OCR_00447] Firestone R. A. (1994). Low-density lipoprotein as a vehicle for targeting antitumor compounds to cancer cells.. Bioconjug Chem.

[OCR_00452] HAVEL R. J., EDER H. A., BRAGDON J. H. (1955). The distribution and chemical composition of ultracentrifugally separated lipoproteins in human serum.. J Clin Invest.

[OCR_00457] Ho Y. K., Smith R. G., Brown M. S., Goldstein J. L. (1978). Low-density lipoprotein (LDL) receptor activity in human acute myelogenous leukemia cells.. Blood.

[OCR_00462] Kahl S. B., Callaway J. C. (1989). New tumor localizers: advances in the use of low density lipoproteins (LDL).. Strahlenther Onkol.

[OCR_00475] Laster B. H., Kahl S. B., Popenoe E. A., Pate D. W., Fairchild R. G. (1991). Biological efficacy of boronated low-density lipoprotein for boron neutron capture therapy as measured in cell culture.. Cancer Res.

[OCR_00482] Masquelier M., Vitols S., Peterson C. (1986). Low-density lipoprotein as a carrier of antitumoral drugs: in vivo fate of drug-human low-density lipoprotein complexes in mice.. Cancer Res.

[OCR_00491] Ponty E., Favre G., Benaniba R., Boneu A., Lucot H., Carton M., Soula G. (1993). Biodistribution study of 99mTc-labeled LDL in B16-melanoma-bearing mice. Visualization of a preferential uptake by the tumor.. Int J Cancer.

[OCR_00497] Rudling M. J., Angelin B., Peterson C. O., Collins V. P. (1990). Low density lipoprotein receptor activity in human intracranial tumors and its relation to the cholesterol requirement.. Cancer Res.

[OCR_00503] Setiawan Y., Rise T., Moore D. E. (1994). Fourier transform infrared (FTIR) spectrometry for the assay of polyhedral boron compounds in plasma and pharmaceutical formulations.. Pharm Res.

[OCR_00513] Tamat S. R., Moore D. E., Allen B. J. (1987). Determination of boron in biological tissues by inductively coupled plasma atomic emission spectrometry.. Anal Chem.

